# Safe and efficient novel approach for non-invasive gene electrotransfer to skin

**DOI:** 10.1038/s41598-018-34968-6

**Published:** 2018-11-15

**Authors:** Lise Pasquet, Sophie Chabot, Elisabeth Bellard, Bostjan Markelc, Marie-Pierre Rols, Jean-Paul Reynes, Gérard Tiraby, Franck Couillaud, Justin Teissie, Muriel Golzio

**Affiliations:** 1 Institut de Pharmacologie et de Biologie Structurale, Université de Toulouse, CNRS, UPS, BP 64182, 205 Route de Narbonne, Toulouse, F-31077 France; 2 Invivogen Cayla SAS, 5 rue Jean Rodier, Zone industrielle de Montaudran, 31400 Toulouse, France; 3 Laboratoire d’Imagerie Moléculaire et Thérapies innovantes en Oncologie (IMOTION) EA 7435, Université de Bordeaux, Bordeaux, France

## Abstract

Gene transfer into cells or tissue by application of electric pulses (i.e. gene electrotransfer (GET)) is a non-viral gene delivery method that is becoming increasingly attractive for clinical applications. In order to make GET progress to wide clinical usage its efficacy needs to be improved and the safety of the method has to be confirmed. Therefore, the aim of our study was to increase GET efficacy in skin, by optimizing electric pulse parameters and the design of electrodes. We evaluated the safety of our novel approach by assaying the thermal stress effect of GET conditions and the biodistribution of a cytokine expressing plasmid. Transfection efficacy of different pulse parameters was determined using two reporter genes encoding for the green fluorescent protein (GFP) and the tdTomato fluorescent protein, respectively. GET was performed using non-invasive contact electrodes immediately after intradermal injection of plasmid DNA into mouse skin. Fluorescence imaging of transfected skin showed that a sophistication in the pulse parameters could be selected to get greater transfection efficacy in comparison to the standard ones. Delivery of electric pulses only mildly induced expression of the heat shock protein Hsp70 in a luminescent reporting transgenic mouse model, demonstrating that there were no drastic stress effects. The plasmid was not detected in other organs and was found only at the site of treatment for a limited period of time. In conclusion, we set up a novel approach for GET combining new electric field parameters with high voltage short pulses and medium voltage long pulses using contact electrodes, to obtain a high expression of both fluorescent reporter and therapeutic genes while showing full safety in living animals.

## Introduction

The skin is not only a physical barrier that shields the body from external agents. This organ is also an attractive target for gene therapy and vaccination, due to its accessibility, large surface area and the presence of numerous immune cells such as Langerhans cells and other dendritic cells that can elicit appropriate immune responses to defend the body^1,2^. Therefore, Bacille Calmette-Guérin and rabies vaccines are acting by the direct injection of using either dead or attenuated virus (or bacteria) or recombinant protein into the dermis for immunization^3^. These conventional immunizations are most of the time disappointing due to their low effect against intracellular pathogens and cancers. This is the result of the lack of cellular responses. New technologies, such as DNA vaccines elicit both broad humoral and cellular high level immune responses^4^. The barrier properties of the skin remains a technical challenge by limiting the penetration of DNA in the skin cells. Results from a direct injection skin DNA immunization are limited. Cutaneous gene therapy need to be efficient, specific and the DNA expression need to be controllable in time. Over the years, several chemical and physical methods have been implemented to enhance skin DNA delivery (see for review^5^). In the field of gene delivery, most researches used the highly effective viral gene transfer. However, this method presents several side effects such as generation of novel infectious agents, immunogenicity of the vector and mutational insertion of viral DNA. In order to avoid these negative effects and still be able to introduce large molecules of DNA there is still a need to develop safe non-viral gene transfer approaches^6^.

Direct *in vivo* application of short high voltage pulses has been shown to permeabilize skin cells. The first *in vivo* electroporation-mediated gene transfer was obtained by Titomirov and colleagues. They transfered an antibiotic resistance gene into the skin of newborn mice. Expression was detected in skin fibroblast extracted from the dermis^7^. Studies showed that gene electrotransfer in the skin affects various cell types such as keratinocytes, adipocytes and fibroblasts as well as Langerhans cells and other mononuclear cells with dendritic processes. The transfected cells appeared to be located in different layers of the skin from the epidermis to the hypodermis through the dermis and as deep as the subcutaneous muscle layer^8–13^. Transfected cells have also been observed in draining lymph nodes^14^. Therefore, skin appeared to be a good target for immunization with an easy access^15–17^. One example of efficient use of skin gene electrotransfer comes from study delivering a plasmid expressing VEGF in an ischemic rat skin flap model to enhance wound healing^18^. The intradermal injection of DNA followed by the skin permeabilization with electric field pulses produce a local treatment dependent on the spatial and temporal distribution of the DNA and the electric field. This treatment has the main advantages of being easy to use, fast, reproducible and safe.

The skin is the first line of defense of the organism against invading antigens due to its high number of professional antigen-presenting cells. Therefore, various antigens were expressed in the skin to elicit a response of this high level of antigen-presenting cells. As in muscle, expression of antigens following skin pDNA GET generates both CD8 and CD4 responses^17^.

Gene electrotransfer (GET) is obtained by a local and controlled injection of a small volume of a plasmid solution followed by electrical voltage pulses delivery between electrodes in contact with the target tissue^19^. GET results from an electrophoretic driven accumulation of the plasmid (pDNA) towards the surface of the target cells, that are electropermeabilized^20,21^. Transfection yield (expression of the protein encoded by the pDNA) is therefore under the control of the transfer across the target cell membrane. The electric parameters that control the pDNA electrophoretic drift in the bulk and the level of membrane permeabilization should be selected to preserve the cell viability. Firstly, the local field applied on a target cell in a tissue is known to be a key parameter to obtain membrane permeabilization but can be destructive^22^. Secondly, the pulse duration plays a complex positive role^23^. Thirdly, the number of repetitive pulses in a train increases the local accumulation of pDNA^24^. Finally, as the electric field is a vector, the direction of the applied field that is under the control of the position of the electrodes is of major importance^25^.

The field distribution in the target tissue depends on its electrical properties but mostly on the design of the electrodes^26^. Skin GET was first obtained by pinching the skin between parallel plate electrodes. This was limited to parts of the skin that were highly flexible to be introduced between the plates. In that case, large skin area could be treated. The resulting field distribution brought the electropermeabilization of the tissue under the skin as predicted by electrical modeling^27,28^. Minimally invasive electrodes were designed with arrays of microelectrodes at high density. They could be perforating the skin to get rid of the insulating barrier played by the stratum corneum (SC). Even though small injuries were induced with a risk of local inflammation, they were proved to be effective for gene transfer and to mediate a host immune response. A low voltage was delivered between each neighboring needle that resulted in a local heterogeneous field. Spots of expression were present only close to the needle electrodes^29–37^. Contact multi electrodes arrays were described by other groups, providing an electric field present across the SC. A conductive gel was added between the tip of each electrode and the skin. Higher voltage to electrode distance ratio were therefore needed to obtain the electrical conductivity across the SC and as result a way to affect the dermis and the epidermis. This technology was observed to be effective for pDNA expression in the skin. Again, as the field in the skin was not homogeneous, spots of pDNA expression were detected in the treated skin. Damages to the skin resulting from the SC alterations were limited by the proper choice of the electrical parameters^9,38–42^. Our group introduced a few years ago another concept of non-invasive electrodes allowing the electrotreatment of a large skin area^43^. These contact electrodes were observed to be highly safe and efficient for electroimmunization^44^.

Protein expression obtained after GET is also dependent on the construct. The size of the plasmid is proven a key element. Mini-circle plasmids were shown to induce a higher rate of protein expression^45^. This was described as a consequence of the damages associated with the transmembrane translocation of the construct^46^. The choice of the promoter played a role in the level of expression and its tissue specificity^47^.

The full nature of the immune and tissue responses to nucleic acids and electrotransfer to the skin has not been addressed. It was observed in muscles that the nature of the inflammatory infiltrate and the kinetics of gene expression were different along electrotransfer by conventional and CpG-free plasmids^48^.

Despite the key advantage of pDNA GET in the skin for vaccination there is always room for improvement especially in regards to GET parameters to limit the targeted tissue to the skin and not to the underlying tissue while keeping an efficient expression. The inflammatory response of the treated tissue must be limited in time and the delivery method needs to be safe. This can be improved by a proper choice of the pDNA construct. The delivery method needs to be safe and locally controlled in order to avoid spreading of the transgene.

In the present study, we analyzed the expression of reporter and therapeutic genes by using a set of non-invasive contact electrodes under different pulsing conditions (duration, voltage, direction). The long-term persistence of a therapeutic plasmid was approached and the thermal and stress effects were evaluated.

## Results

### Optimization of the electric parameters

**Figure 1:**
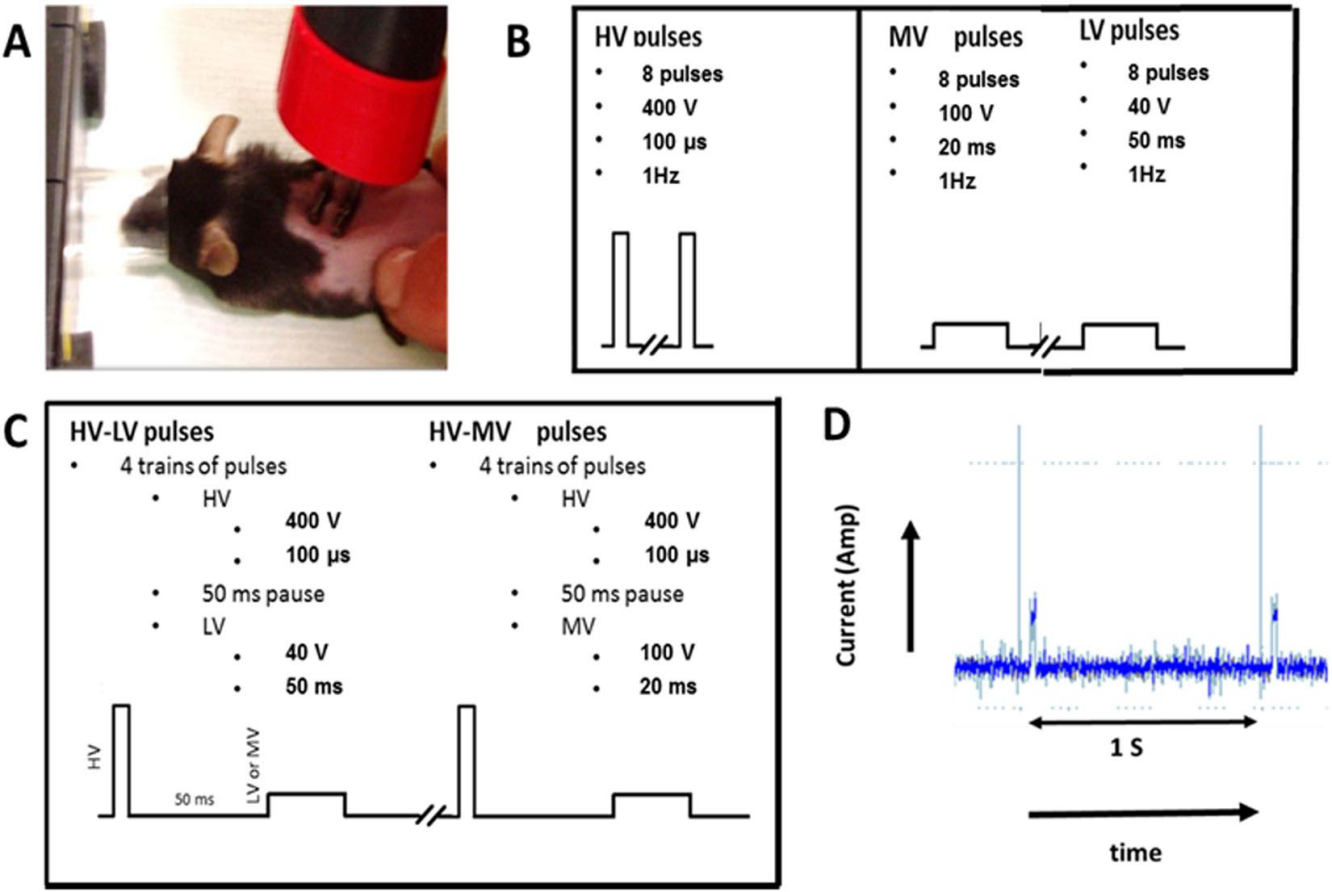
Gene electrotransfer *in vivo*. (**A**) Electrode set-up applied on the mouse skin. (**B**) Simple electric parameters and corresponding voltage profiles. (**C**) Combination of electric parameters and corresponding voltage profiles. (**D**) On line monitoring of the current associated to the HV-MV pulse train (2 successive trains are displayed). The vertical arrow is 0.5 Amp

In order to increase the transfection yield of plasmid in the skin and therefore the expression of this plasmid, we first evaluated the impact of a large set of pulsing conditions all applied using contact wire electrodes. High Voltage (HV) consists in 100 µs square waved pulses of 400 V, Low Voltage (LV) were 50 ms square waved pulses of 40 V and Medium Voltage (MV) were 20 ms square waved pulses of 100 V (Fig. 1). The intermediate time delay was 50 ms. These parameters were selected to ensure that no tissue damage should result from the treatment and keeping in mind that High long electric field pulsing conditions were shown to induce a local burning and tissue damage in the inter-electrode space^44^.

**Figure 2:**
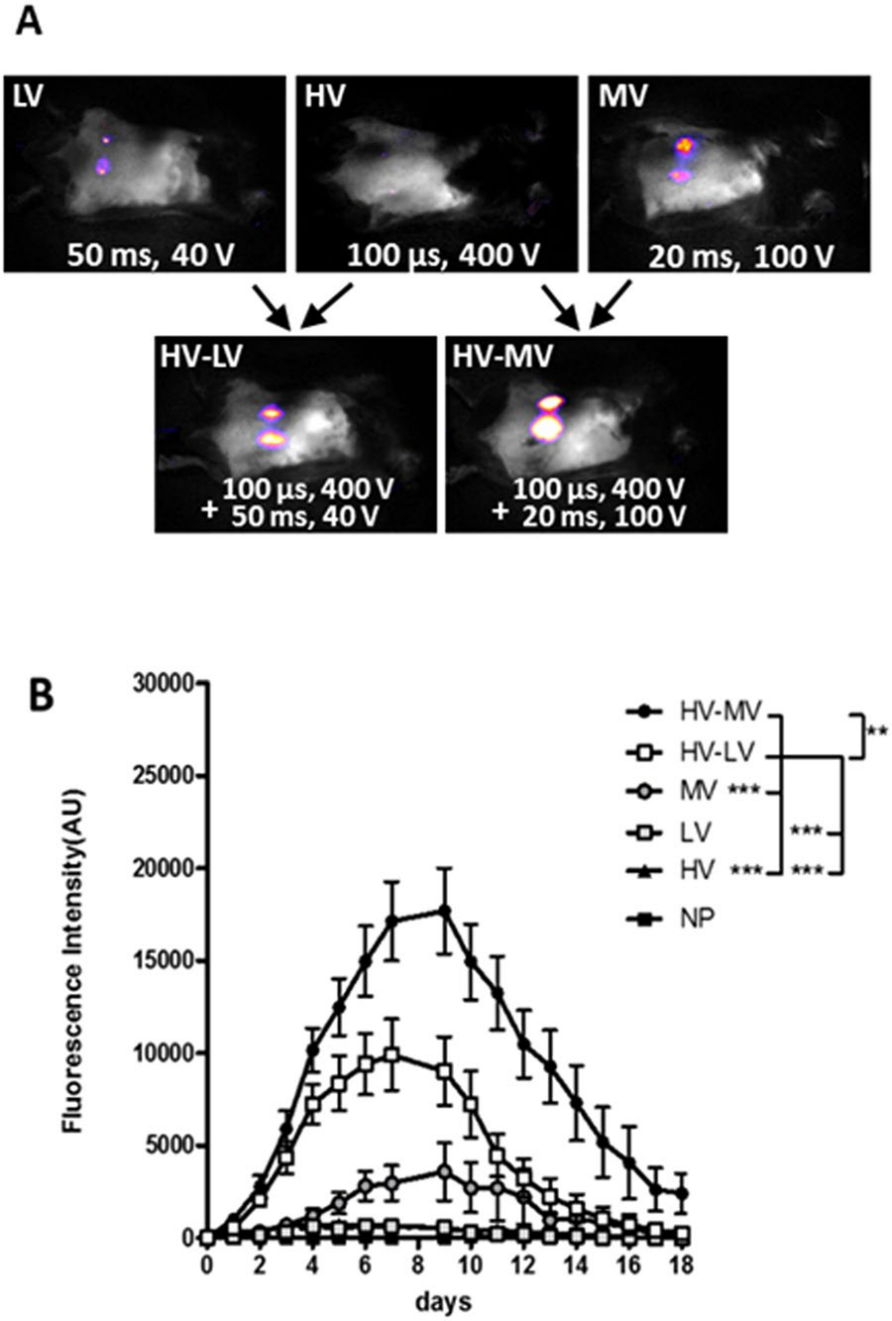
Evaluation of electrical parameters settings for cutaneous transgenic expression. Plasmid DNA (25 µg) encoding the fluorescent protein tdTomato were intradermally injected in the back of anesthetized C57Bl/6 mice and the electrotransfer was performed. Mice received either 8 pulses of LV (50 ms, 40 V), HV (100 µs, 400 V) or MV (20 ms, 100 V) or 4 trains of pulses of combination of either HV-LV (100 µs, 400 V + 50 ms, 40 V) or HV-MV (100 µs, 400 V + 20 ms, 100 V)). Two sites of injection were treated with the same electrical parameters. Images were acquired every day after GET with fluorescence macroscopy and the mean fluorescence intensity of tdTomato was determined with ImageJ software. (**A**) Representative images of tdTomato fluorescence (in pseudo color) at day 1. (**B**) tdTomato expression was followed over time by non-invasive fluorescence microscopy. Values are means ± s.e.m., n = 11–14 except for non-pulsed mice (NP) where n = 3. **P < 0.01, ***P < 0.001 (Two-way ANOVA analysis).

We observed that tdTomato expression, when detectable, was always located between the electrodes. LV and HV parameters used alone induced an almost undetectable expression of the protein whereas the application of MV parameters induced a consistent expression of the tdTomato protein. This condition induced a maximum of expression by 7 to 10 days after treatment and then a decrease in expression until being undetectable by day 18. The association of HV and LV parameters induced an improved tdTomato expression that was further increased using the combination of HV and MV parameters. In both cases, the maximum of expression was observed 8 days after treatment (Fig. 2). However, while the HV-LV parameter induced a transitory expression that became undetectable by day 18, the HV-MV parameters induced a sustained expression over 18 days easily detectable by imaging. Therefore, we selected the HV-MV parameters for further optimization studies. Moreover, imaging of the animal in the days following the delivery showed that the expression of the tdTomato measured by the evaluation of the fluorescence intensity was homogeneous in the gap between the electrodes and was not a mosaic of spots (S2). This was confirmative of the mathematical prediction under Comsol simulation already published where it was obtained a homogeneous field distribution in the space between the parallel electrodes (plate as well as contact wire)^22^.

### Effect of the electrode design on gene expression

After intradermal injection, the papule formed in the skin was soft enough to be placed between plate electrodes. A train of 4 HV-MV pulses was used as it was shown to be highly efficient when using the contact electrodes. Plate electrodes were known to deliver a homogeneous field on the sample present between the electrodes. Treated skin surface was 0.4 cm^2^ for two types of electrodes. Therefore, the same settings (voltage to electrode gap, duration delay) were kept.

**Figure 3:**
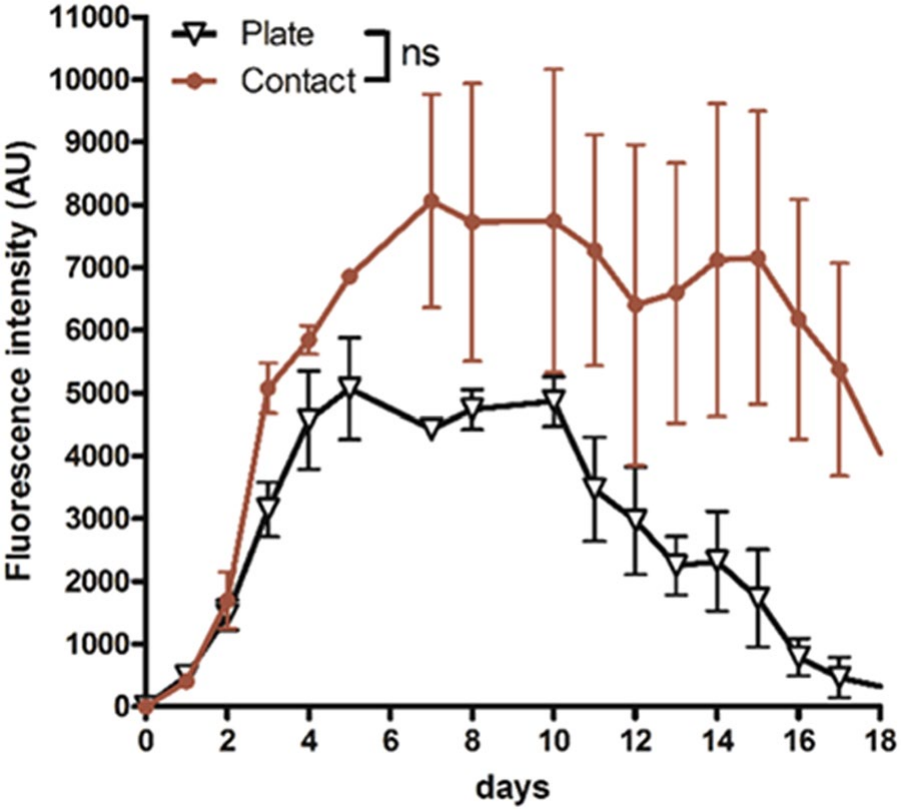
Optimization of the HV-MV parameters for cutaneous transgenic expression. Plasmid DNA (25 µg) encoding the fluorescent protein tdTomato were intradermally injected in the back of anesthetized C57Bl/6 mice and the electrotransfer was performed using the HV-MV (100 µs, 400 V + 20 ms, 100 V) parameters. Two sites of injection were treated with the same electrical parameters. The tdTomato expression was followed over time by non-invasive fluorescence microscopy. Electrotransfer was performed either with plate or contact electrodes (4 trains of HV-MV pulses Values are means ± s.e.m., n = 3. ns = non-significant result (Two-way ANOVA analysis).

As shown on Fig. 3, the results obtained with plate electrodes were similar to those obtained with the contact electrodes. The level of tdTomato fluorescence as well as the time required to detect the expression and the pattern of expression were similar with one or the other set of electrodes. However, tdTomato expression appeared more stable in time when using contact electrodes compared to plate electrodes.

Contact electrodes were selected for the development of the study (more user friendly, as they are just placed on the top of the skin around the papule).

### Effect of the number of train of pulses

We described in Fig. 2 the efficiency of the HV-MV parameters. In this original setting 2 successive pulses with different magnitudes and durations are delivered repetitively during a train. Previously the number of double pulses (n) in a train was set to 4 and all were applied in a unipolar direction. We evaluated if the number of pulses was affecting the expression (in magnitude and duration). As determined previously, mice were injected with 25 µg of plasmid tdTomato and treated with different numbers of pulses. The tissue integrity was also closely observed.

**Figure 4:**
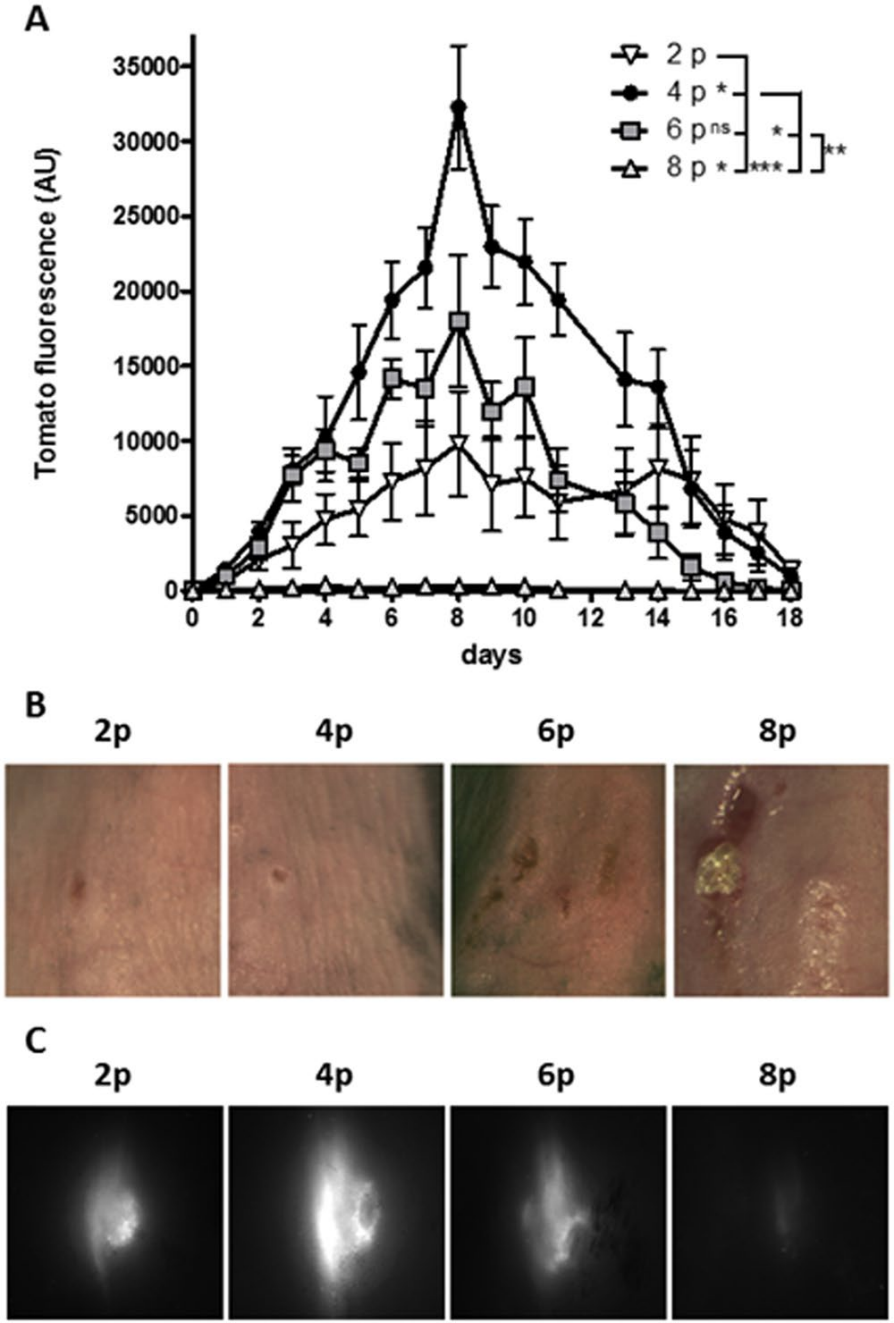
Optimization of the HV-MV parameters for cutaneous transgenic expression. 25 µg of plasmid DNA coding the fluorescent protein tdTomato were intradermally injected in the back of anesthetized C57Bl/6 mice and the electrotransfer was performed using the HV-MV (100 µs, 400 V + 20 ms, 100 V) parameters. (**A**) Mice received 2, 4, 6 or 8 trains of pulses. Two sites of injection were treated with the same electrical parameters. tdTomato expression was followed over time by non-invasive fluorescence microscopy. Values are means ± s.e.m., n = 4. *P < 0.05, **P < 0.01, ***P < 0.001, ns = non-significant result (Two-way ANOVA analysis). (**B**) Representative pictures of electrotransferred skin at day 1. (**C**) Representative images of tdTomato fluorescence at day 1. Values are means ± s.e.m., n = 3. ns = non-significant result (Two-way ANOVA analysis).

Two trains of pulses were sufficient to detect a significant expression of the tdTomato protein. The application of 4 trains of pulses induced a 3 to 4-fold higher expression than 2 trains of pulses. The application of 6 trains of pulses induced an intermediate expression between the ones obtained with 2 trains and 4 trains suggesting a limiting effect of the number of pulses that can be applied. This was confirmed by the absence of tdTomato detection with 8 trains of pulses (Fig. 4A). The fluorescence was always detected as a homogeneous spot but with a larger intensity with 4 trains (Fig. 4C). The time course of expression was similar whatever the number of trains. The highest expression was obtained on day 8 after the treatment. Direct observation of the skin showed that it was partly damaged with 6 trains of pulses and largely injured with 8 trains (Fig. 4B). These damages may have induced cell death that can explain the observed decrease of tdTomato expression with the higher number of train of pulses. Therefore, 4 trains of HV-MV pulses were selected as the best choice.

### Effect of polarity inversion

The transfer of pDNA along GET is supported by electric forces. They are vectors and the direction of the field can be considered as a leading factor in gene electrotransfer. It was indeed observed *in vitro* that the pattern of DNA interaction with cell surfaces was dependent on the field orientation^25^. We showed that 4 trains of HV-MV pulses delivered in unipolar direction were highly effective for expression and did not produce skin damage. In the following set of experiments, the direction of the field was inverted after each train by changing the polarity of the electrodes. The energy was the same whatever the polarity settings bringing the same Joule heating.

**Figure 5:**
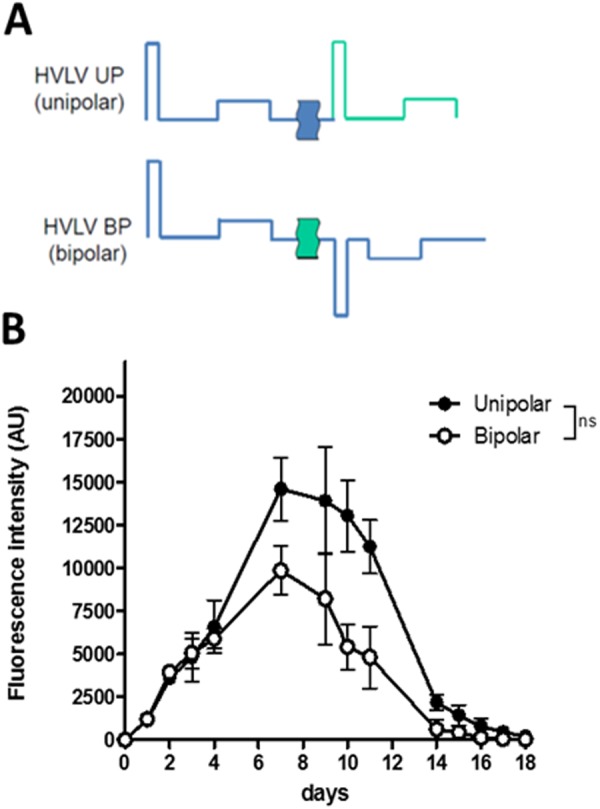
Time dependence of the voltage present between the electrodes under the unipolar and bipolar settings. (**A**) Electric parameters and corresponding voltage profiles. A 1 s delay was present between each HV pulse. This was obtained by the settings of the B10 electropulsator keeping the 1 Hz frequency for the delivery of trains. (**B**) tdTomato expression was followed over time by non-invasive fluorescence microscopy. Values are means ± s.e.m., n = 4. ns = non-significant result (Two-way ANOVA analysis).

The bipolarity of the pulses did not bring any improvement in expression either in the level or in the duration of the tdTomato expression (Fig. 5). The mean fluorescence was maximal at day 8 and then decreased. A slight (statistically non-significant) decrease was present. No skin damage was observed whatever the polarity settings. A similar conclusion was recently obtained with a peritumoral gene electrotransfer with plate electrodes used to apply 600 V/cm pulses at the frequency of 1 or 2 Hz^49^. Therefore, 4 unipolar trains of HV-MV pulses were selected for the following experiments.

### Gender effect

**Figure 6:**
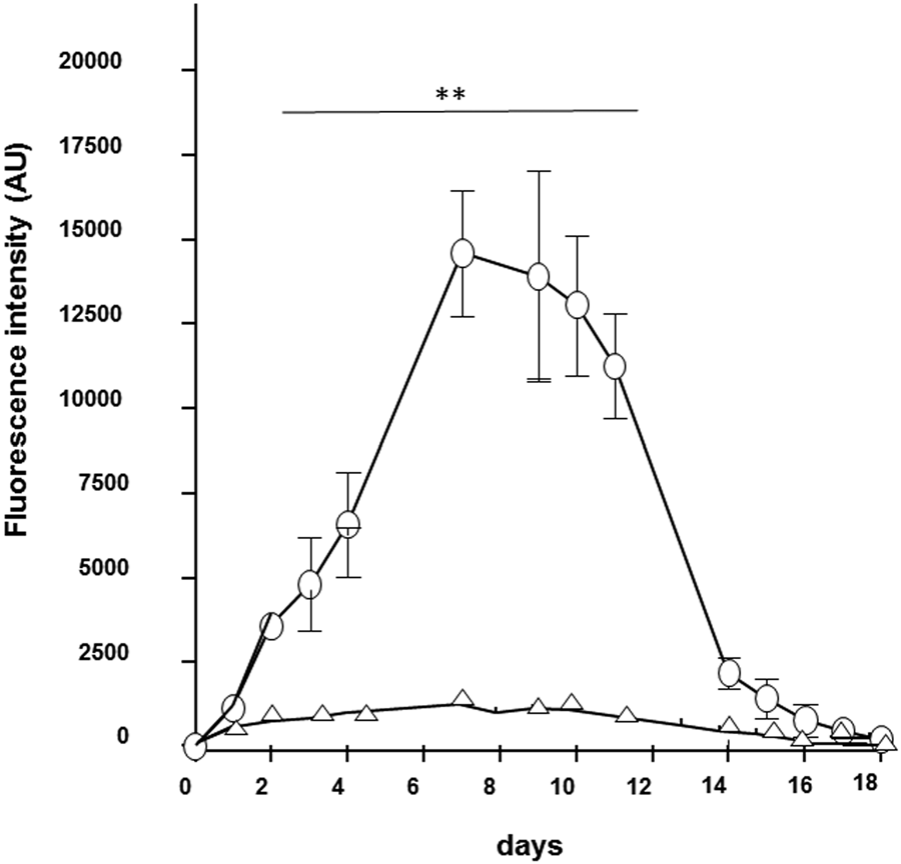
Expression levels for dtTomato for male and female mice. 25 µg of plasmid DNA coding the fluorescent protein tdTomato were intradermally injected in the back of anesthetized C57Bl/6 mice (female ○, male Δ) and the electrotransfer was performed using the HV-MV (100 µs, 400 V + 20 ms, 100 V) parameters. tdTomato expression was followed over time by non-invasive fluorescence microscopy. Values are means ± s.e.m., n = 4. **P < 0.01 (Two-way ANOVA analysis).

Our assays were performed on both female and male animals. A large difference in the level of expression was observed for the two genders (Fig. 6). The expression of dtTomato was observed during 20 days in both cases but the fluorescence emission was 15 times lower for the males. We noticed that the physiology of the skin was different. For males, the skin was thicker and more difficult to inject. A part of the plasmid solution may not be properly injected; it could explain the lower level of expression in this figure. Another problem is in the detection of the fluorescence signal as the increase in light scattering due to the thickness and composition of the skin of male mice could lead to a decrease in the incident light at the target and a loss in the emitted light collection.

### Evaluation of Hsp 70 (Hspa1b) expression after GET

**Figure 7:**
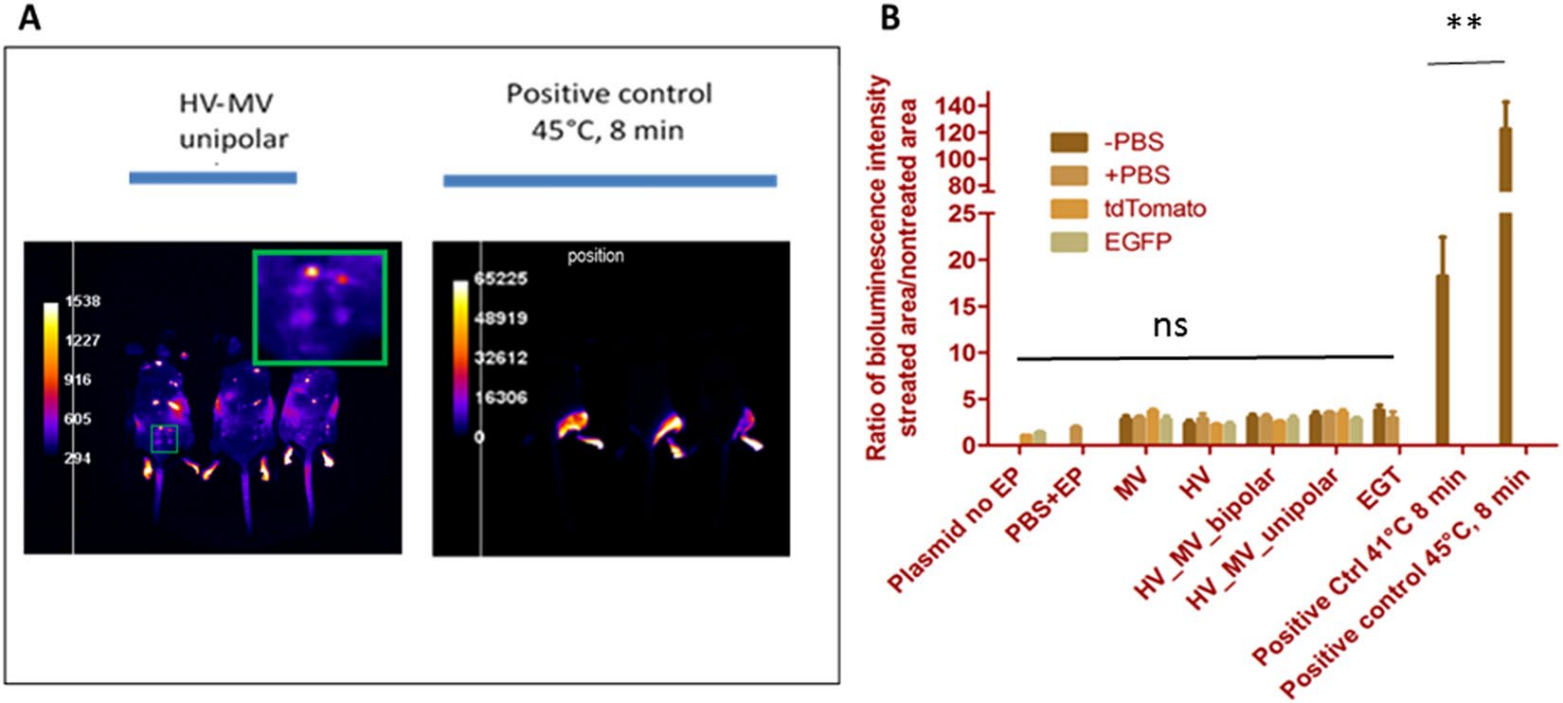
Bioluminescence imaging of EP-induced expression of LucF in transgenic Hsp70 (Hspa1b) LucF mice. Luciferin (30 mg/mouse) was injected intraperitoneally 5 min before imaging (**A**) Images were acquired 6 h after delivery of electric pulses. (**B**) Quantification of LucF expression in transgenic mice after EP presented as the fold increase of the signal at the position of electrodes compared to the detected signal in non-treated skin. Groups on the left graph. −PBS: only EP, +PBS: 25 µl of PBS injected intradermally + EP, tdTomato: 25 µl (1 µg/µl) of plasmid tdTomato injected intradermally + EP, EGFP: 25 µl (1 µg/µl) of plasmid EGFP injected intradermally + EP. EP conditions (HV, MV and HV-MV) are those defined in Fig. 1. EGT 25 µl (1 µg/µl) of plasmid EGFP injected intradermally + 8 pulses of 5 ms at 240 V.Positive controls were water bath at designated temperature for 8 min.

Potential thermal damages might be induced by the electrical treatment^50,51^. A positive contribution was shown to be brought by a controlled heating of the skin where the electrotransfer was delivered^52,53^. The local heating of the skin was investigated as a potential parameter in the control of the gene expression. 4 trains of HV-MV pulses were selected as the best choice. No skin damage was induced (Fig. 4). The pulse associated Joule heating was assessed *in vivo* by bioluminescence imaging using a transgenic mouse strain expressing the luciferase reporter gene under transcriptional control of a thermosensitive promoter. Under the HV-MV conditions, no increase in the luciferase expression was detected 6 h after delivery of electric pulses, whatever the pulsing conditions or the injected vehicle (PBS, tdTomato or GFP plasmid) (Fig. 7).

### Evaluation of local expression and secretion of IL-12 after skin pIL-12 GET

**Figure 8:**
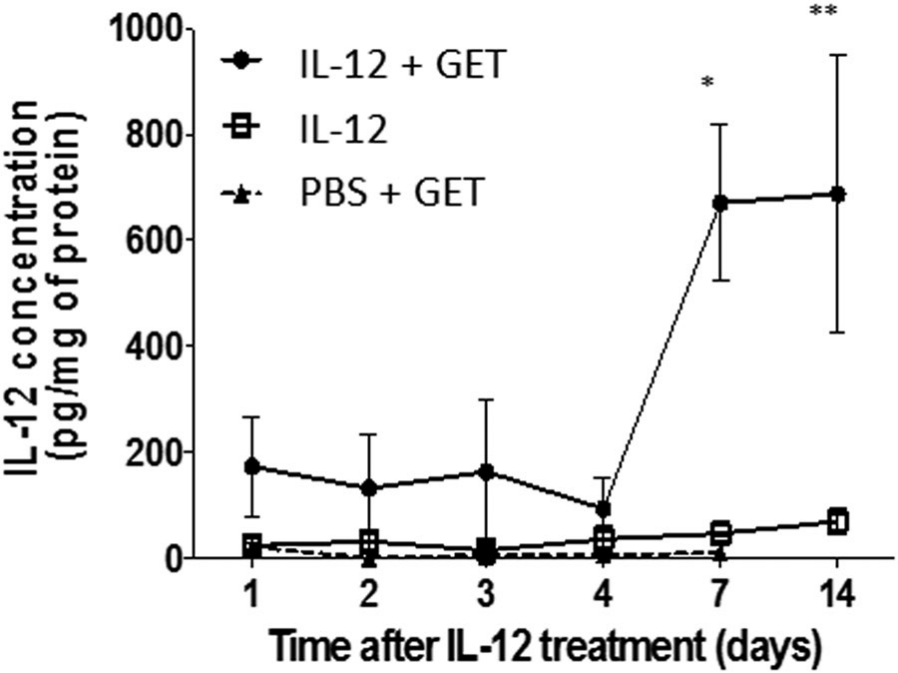
pIL-12 GET induces expression and secretion of IL-12. Mice were intradermally injected with 25 µg of pIL-12 in 20 µl PBS, or with 20 µl of PBS alone in one flank and were submitted to HV-MV electrical treatment. The contralateral flank from pIL-12 injected mice was non-injected and non-pulsed to be used as an internal control. Mice were sacrificed from 1 to 14 days after treatment and skins from each flank were harvested and cultured overnight in complete medium 5% CO_2_, 37 °C. IL-12 content was determined by ELISA in the cultured supernatant of pIL-12 treated skin (IL-12 + GET), of PBS treated skin (PBS + GET) and in contralateral untreated skin from pIL-12 GET treated mice (IL-12). (n = 5, 3 independent experiments). Statistical analysis: 2way Anova, *p < 0.05, **p < 0.005.

The possibility to express ectopically a tumor antigen or a stimulatory protein of the immune system could help in the eradication of primary tumors and/or metastasis is of great interest. We decided to evaluate the efficiency of our newly developed electrical parameters on the expression of a plasmid encoding the cytokine IL-12. IL-12 plays a central role on the activation of the immune system orienting the anti-tumor immune response toward a cytotoxic Th1-like immune response. In order to determine the efficiency of *in vivo* pIL-12 gene-electrotransfer (pIL-12 GET), 25 µg of pIL-12 or a same volume of PBS were intra-dermally injected in the left flank of C57Bl/6 mice. Rapidly within a few seconds, the skin was treated under HV-MV electrical parameters applied using contact electrodes (4 unipolar trains). The right side was not injected and not treated to be used as a control. Treated (left flank) and untreated (right flank of the same mice) skin biopsies of pIL-12-GET and PBS-GET treated mice were harvested between 1 and 14 days after treatment and incubated in complete medium for 24 h at 37 °C. IL-12 production was determined by ELISA in the skin cultured supernatant (Fig. 8). Small amount of IL-12 were detected in the supernatant of PBS treated skin (10 pg/ml/mg of protein). However, pIL-12 GET induces a significant increase in IL-12 secretion (173.7 ± 92.97 pg/mg of total proteins at day 1), reaching a peak at day 7 (670 ± 147.91 pg/mg of total proteins) which was maintained up to day 14 post-treatment. These results correlated with the expression of the tdTomato protein used to set up the GET electrical parameters. IL-12 production in the contralateral skin was significantly lower (70.0 ± 23.29 pg/ml at day 14) suggesting a local production of IL-12 at the treated site. Therefore, pIL-12-GET induced an efficient transfection of skin cells resulting in a local expression of the plasmid and thus a local secretion of IL-12 by transfected cells.

### Evaluation of plasmid biodistribution

The transfer of pDNA-GET into the clinic require a thorough evaluation of the biodistribution of exogenous DNA in the body. Mice (male and female) treated with pIL-12-GET were sacrificed at various time points and the expression of the plasmid was searched in lungs, liver, spleen, kidneys, lymph nodes, skin and gonads by Q-PCR (Table 1). No plasmid was amplified in gonads, kidneys, liver, lungs, lymph nodes and spleen for male and female mice at d15 and d49 in the control group and in the GET group. No plasmid was detected in skin of male and female mice in control group at d15 and d49. Results in skin of male and female mice from GET treated group showed a quantified presence of plasmid at d15 and d49 but the amount of plasmid per microgram of tissue was 20 times less at d49 than at d15. These results suggest the safety of the treatment.

**Table 1:** Evaluation of plasmid dissemination.

	Organs		Lungs	Liver	spleen	kidneys	Lymph nodes	skin	gonads
								pg/μg Tissue DNA	
	Days after IL12 Injection								
group control	D15		no	no	no	no	no	no	no
	D49		no	no	no	no	no	no	no
group GET	D15		no	no	no	no	no	0.8	no
	D49		no	no	no	no	no	0.045	no

4 male and 4 female mice were sacrificed for the treated group, 2 male and 2 female mice for the vehicle group. Lungs, liver, spleen, kidneys, lymph nodes, skin and gonads were collected and the presence of the plasmid was analyzed by Q-PCR at day 15 and day 49 after pIL-12 electrotransfer.

## Discussion

Our GET protocol induces a local transient expression of the plasmid in the skin area that was submitted to the combined electric pulses (Fig. 2). It was previously shown, that the combination of a high field short pulse (1000 V/cm, 100 µs) (to induce the electropermeabilization of a large cell surface) and of a low field long pulse (200 V/cm, 400 ms) (to increase the electrophoretic accumulation of plasmids on the cell surface) allowed to obtain a high level of gene expression after GET^11^. In that study, the authors used parallel, stainless-steel plate electrodes, pinching the skin between the two electrodes to delivers the electric pulses. However, the definition of the proper settings of the electric parameters to obtain an efficient GET are dependent on the geometry of the electrodes and the target tissue organization. We observed indeed that the proper setting with our contact electrodes was with a higher voltage to electrode gap ratio in the HV-MV step than described in previous works where arrays of small electrodes were used^34,38,41^.

As expected from the field distribution associated with the contact electrodes, the level of gene expression was homogeneous in the skin area present between the two electrodes where the plasmid solution was injected (Supplementary Fig. 2 and Fig. 3). The expression was first detected in the upper layers of the skin and then sustained for a long time most probably due to a transfection of the muscle layer below the hypodermis. Simulations predicted that the field should be high enough for transfection down to the subcutaneous muscles^43^. The lower level of expression obtained with the more classical plate parallel electrodes where the skin is placed between the two electrodes can be associated with a less suited distribution of the field in the tissue. With plate electrodes a large part of the field is affecting the tissue under the skin while a part of the skin is not in contact with the electrodes and is therefore, poorly affected by the field^28^. This is in line with other studies showing that contact electrodes (multi-array) are more suitable for transfer to the skin^29–37^. The geometry of the contact electrodes we used is user friendly while allowing treating homogeneously a large part of the skin in one single shot of a pulse train (Fig. 3). Another advantage of the contact electrodes is that expression is present in all the space between the two electrodes giving a high total level of expression while a patterned expression is observed with multi-array devices due their technology and the associated heterogeneous field distribution in the skin^31,32,42^.

A cumulative effect of the number of pulses delivered was observed on the transfection efficiency with the lowest number of pulses (Fig. 4) before reaching a point where the advantages of the cell electro-transfection are overcome by the side effects, i.e., skin burning. These observations confirmed *in vitro* results showing that the increase of pDNA transfer due to the increased pDNA accumulation at the cell surface is balanced by the toxic effects of the pulses on cells. This toxicity prevents the expression of the transferred copies. A similar effect was described in a previous report using this electrode technology^44^.

GET is strongly controlled by the polarity of the pulses along a train. Polarity inversion was shown to induce positive additive effects *in vitro*^25^ and *in vivo* on muscles^54^. In the skin, such a positive effect was not observed (Fig. 5). The delay in the pulse inversion (1 s) was long enough to avoid a negative effect obtained with very short delay *in vitro*^55^. The slightly negative effect may be linked to a more destructive effect of the electric field with polarity inversion on the electro-transfected cells.

Expression is detected in a Gender dependent way. A higher fluorescence emission is detected on transfected females. A trivial technical reason is the control of *in vivo* fluorescence imaging by the optical properties of the skin. This is known to be strongly gender dependent^56,57^. This appears to be associated to the differences in the physiology of the skin. The dorsal skin in males is known to be thicker than in females but the epidermis and hypodermis thicknesses were larger in females. The hypodermis is indeed 10 times larger in females. The adipose layer is more abundant in female mice as compared to males. This supports a conclusion where the expression due to GET after ID injection of the plasma is mostly present in the hypodermis and in the fatty tissues. The adipocytes were recently shown to be a target for GET in the skin^58^.

Electro-transfection might induce some side effects by locally increasing the temperature of the tissue. We assess the effect by using HSP70-luc reporter mice. In this model, a macroscopic temperature rise was necessary to induce HSP70 gene expression but the HSP70 promotor can also be activated in response to other stress origins^59^ inducing intracellular protein denaturation. Stressors include oxidative stress^60^ present in electropermeabilization^61^, or energy depletion^62^, or a direct effect of cell electropermeabilization^63^. Activation of the HSP70 response was not observed by the bioluminescence studies (Fig. 8). Therefore, this lack of responses in the bioluminescence imaging is one more piece of evidence that our GET strategy is not stressing the tissue. A similar conclusion was reported for GET in muscles^64^.

The study with a more physiological relevant plasmid coding for IL12 confirmed the efficacy of the transfection and provided a key information on the safety of the transfer of a gene to the skin (Fig. 8). The design of a IL12 coding plasmid is known to be a decisive parameter for cancer immunotherapy^65^. A CpG free form was selected as those bacterial dinucleotides are immunostimulator motives decreasing the duration of gene expression. IL-12 expression is an effective immunotherapy approach against melanoma^66,67^. With this optimized protocol and the CpG free design of the plasmid construct, the plasmid can be detected only at the site of injection and pulse delivery (Table 1) without affecting other organs. Furthermore, we observed that the plasmid remained present in the skin only during a limited period as on day 49 after the transfer, the DNA that was present was at the limit of detection of the very sensitive PCR Assay. Interestingly, IL-12 is detected as soon as day 1 post-transfection only at the site of transfection and the production increases greatly by day 7 post-treatment. This time delay could correlate with a local production by activated immune cells present to the site of transfection.

## Conclusion

The present approach brings a very user-friendly approach for GET targeted to the skin. A large surface of the skin can be easily homogeneously treated in a short time giving a high level of expression. Successive treatments of different skin areas on the same animal can be easily performed by moving the electrodes to different places where pDNA ID injection was performed. This is fast as the pulse delivery lasts only 3 s. Gene expression would be enhanced by increasing the size of the treatment area^39^. No skin damage was observed. Expression was long lived. The plasmid was detected only at the site of treatment; all other organs remained not affected proving the safety of the protocol for gene delivery.

## Materials and Methods

### Mice

Female C57Bl/6 mice, 6–9 weeks old were obtained from Janvier Labs (Le Genest St. Isle, Saint Berthevin, France). NLF-1 mice contained a transgene that allows firefly luciferase expression under control of the thermo-inducible heat-shock protein (Hsp70) promoter 1B (Hspa1b)^48^. Animal studies were conducted in accordance with the principles and procedures outlined by the European convention for the protection of vertebrate animals used for experimentation. Experiments dealing with the optimization of electric parameters and the IL-12 expression were approved by the IPBS ethics committee (n°20111028/151) and by the ethical commitee “CE001” of the Ministère de l’Enseignement Supérieur, de la Recherche et de l’Innovation (MESRI) (n°01467.02), respectively.

### Plasmids

pCMV-EGFP-C1 a 4.7-Kb plasmid DNA encoding GFP, and pCMV-tdTomato Vector a 5.4 kb plasmid DNA coding for tdTomato fluorescent protein (both from Clontech, Mountain View, CA), were amplified in Escherichia coli DH5α and purified with the Maxiprep DNA Purification System (Qiagen, Germany) according to the manufacturer’s protocol.

pCpGfree-mIL12 (p35p40) was a 4655-bp plasmid encoding mouse IL-12 cytokine was provided by Invivogen (Toulouse, France).

Plasmids were kept in PBS at a concentration of 1 µg/µL and stored at −20 °C.

### Intra-dermal DNA injection and electric pulses delivery

Hair on the back was removed with a hair removal lotion (Veet, France) 2 days before each electro-transfection. Animals were kept under isoflurane/air anesthesia during the whole procedure. The mice were injected in two sites with 25 µg of plasmid in 25 µl PBS by intra-dermal (ID) route using a 300 µl syringe with a 29 G needle (Terumo, France). Following ID injection of plasmid DNA, an electrical field pulse was applied on each injection site with 10 mm long × 1 mm diameter contact wire electrodes (Fig. 1A). The distance between the electrodes (center to center) was 4 mm. Conducting paste (Comepa, St Denis, France) was used to ensure good electrical contact between the electrodes and the skin surface. Non-invasive wire contact electrodes were designed to focus the field in the tissue layer close under the skin between the wires^42^. Treated skin surface was 0.5 cm^2^. Different square wave (unipolar or bipolar) pulses presented in Fig. 1B,C were delivered thanks to the β-tech pulse generator ELECTRO cell B10 (Betatech, St Orens, France). Pulses in the train were applied at the frequency of 1 Hz. The proper delivery of the pulses from the pulse generator was monitored on-line on the touch screen. A current-follower (Chauvin Arnoux, Paris, France) connected to a digitizer (Picoscope, St Neots, UK) was used to register the delivered current profiles (Fig. 1D).

Transfer by plate electrodes was performed as previously described^12^ and was obtained with B10 pulse generator using stainless steel, flat, parallel (4 mm gap) electrodes (IGEA, Carpi, Italy). The skin, where the plasmid injection was performed was squeezed between the two plate electrodes using Echogel to obtain a good electrical contact.

### *In vivo* fluorescence optical imaging of reporter gene

Visualization of fluorescent protein expression was followed *in vivo* over several weeks after gene electrotransfer. Animals were kept under isoflurane anesthesia during the observation. Macrofluo microscope (Leica, Wetzlar, Germany) was equipped with a cooled CCD camera (Roper Coolsnap HQ, Photometrics, Tucson, AZ) using the 0.57 magnification. The exposure time was set at 1 s with no binning. Color imaging was obtained by use of CRI Micro*Color 2 Liquid Crystal Technology. The fluorescence excitation was obtained with an EL6000 light source (Leica, Wetzlar, Germany) and either the L5 (λex = 480/40 nm, λem = 527/30 nm) or the ET mCH/TR (λex = 560/40 nm, λem = 630/75 nm) filter sets (Chroma technology, Rockingham, USA) for GFP and tdTomato observation, respectively. This procedure allows analysis of vector expression on the same animal during several weeks. tdTomato plasmid codes for a protein with an emission in the red (wavelength longer than 630 nm). Compared with GFP, this expression is more easily detected as its spectral range is in skin optical window, where light scattering and absorption are limited and do not alter the detection of the emission. Thus, it was used for most experiments. For images acquisition and quantification, the MetaVue5.2 software (Universal, Downingtown, PA, USA) was used. Images were processed for contrast and brightness. High-resolution images of 1392 × 1040 pixels were captured directly on a Dell PC.

### Bioluminescence imaging and measurements

Bioluminescence allowed for non-invasive spatiotemporal follow-up of transgene expression. The NLF-1 mice were used to follow the heat shock response by *in vivo* imaging of luciferase reporter protein expression. Thermo-induced luciferase expression in transgenic mice was followed by bioluminescence. Thermal stress positive control was delivered by heating the leg of the mouse in a bath of water between 39 °C and 45 °C for 8 min and luciferase activity was measured in anesthetized live animals as a measure of reporter gene transcription (S1)^51^. The bioluminescent imaging system consists of a cooled charge-coupled-device camera (Andor iKon M, Belfast, UK) mounted in a light-tight specimen chamber (Photek, UK) fitted with a light-emitting diode, a Schneider objective VIS-NIR (Cinegon 1.4/12-0515, Germany) and a heating blanket (Harvard Apparatus, USA).Intraperitoneal injection of luciferin (3 mg/mouse) in 100 µl of PBS was followed by a lag of 5 min to get a homogeneous diffusion of the substrate before starting the imaging. The *in vivo* bioluminescence imaging was performed 5 min after injection. Images were acquired with Solis acquisition software (Andor technology). We did a time series of up to 4–5 images with an acquisition time of 5 minutes per time point. The first image (5–10 min) was the one with the higher intensity. Imaging was performed 6 h post heating or pulses as previously established^50^. An image of the light emitted by the mice was captured by using an integration time of 5 minutes. Through the use of Image J software (NIH, Bethesda, US), the luminescent image was presented as a false-color image superimposed on the grayscale reference image. The image-processing component of the software calculated the total pixel values (in Relative Light Units [RLU]) from the luminescent images of the heat treated wound area.

### Determination of cytokine content in supernatant

To measure the secretion of IL-12 in tissues, 25 µg of IL-12 plasmid were injected as described above in the dermis of the left part of the back of shaved mice and gene electrotransfer (GET) was applied using the HV-MV parameters. Control mice were injected with PBS and submitted to the same GET protocol. Treatment areas were clearly identified and harvested as well as contralateral untreated skin from pIL-12 treated mice at day 1, d2, d3, d4, d7 and d14 after treatment. Samples were incubated in 2 mL culture medium (Dulbecco’s Modified Eagle Medium with 4.5 g/l D-Glucose and L-Glutamine (DMEM; Gibco/ Life technology) supplemented with 10% fetal bovine serum (Sigma-Aldrich, St Louis, MO) and the antibiotics penicillin (100 U/ml) and streptomycin (100 U/ml) (Gibson/ Life technology)) at 37 °C, 5% CO2 for 24 h. Supernatant were collected and preserved at −80 °C. Protein mass of skin samples was determined as previously described^49^. Briefly, skin samples were weighted, and 50 µl of lysis buffer (0.1% Igepal (Sigma, St Quentin Falavier, France), 1 mM PMSF (Sigma, St Quentin Falavier, France), 1x Protease Cocktail inhibitor (Sigma) for 10 mg of tissue were added. Skin samples were homogenized using an electrical homogenizer (Ultraturrax IKA, Staufen, Germany). After an incubation of 20 min on ice, suspensions were centrifuged 15 min at 20 000 g at 4 °C. Protein concentration was determined by a Bradford Assay (Bio-Rad, Hercules, CA). IL-12 content in the culture supernatant was determined by ELISA according to the manufacturer protocol (Biolegend ELISA MAX Deluxe Set, Biolegend, San Diego, CA).

### Biodistribution

At d15 and d49 after GET with pIL12, 4 male and 4 female mice were sacrificed for the treated group, 2 male and 2 female mice for the vehicle group. Lungs, liver, spleen, kidneys, lymph nodes, skin and gonads were collected. Each organ was frozen at −80 °C. The samples were sent to C.RIS Pharma (St. Malo, France), for the analysis of the biodistribution by Q-PCR using specific primers for the plasmid: mIL12-FW2 (5′->3′): (20 mer) (Tm = 60.34): 5′ ATCAACAGGGTGATGGGCTA 3′and mIL12-RV2 (5′->3′): (20 mer) (Tm = 59.98): 5′ CATCTTCTTCAGGCGTGTCA 3′. Organs received at −80 °C were homogenized, DNA was extracted and quantified. Q-PCR reactions were performed on 125 ng of DNA in order to amplify the plasmid (test item), if present, and quantify it according to the equation of standard curve.

A standard curve with 8 concentrations (from 0.8 ng to 50 ag) of CpG free mIL12 plasmid in water was assayed for each run (1 run per organ). The two lower concentrations (50 and 80 ag) were not used for the equation calculation but were used to determine the limit of detection (LLOD).

### Statistical analyses

Quantitative data (presented as means ± s.e.m.) were analyzed with Prism 4 software (GraphPad, San Diego, CA). Before carrying out statistical tests, we determined whether the data were normally distributed and evaluated their variance. We then carried out appropriate test as indicated. For *in vivo* time-course experiments, we used two-way ANOVA analysis. We report the actual P-value for each test. P < 0.05 was considered statistically significant.

## Electronic supplementary material

Supplementary data
