# Supplementary material for: Safe and efficient novel approach for non-invasive gene electrotransfer to skin
Source: Sci Rep. 2018 Nov 15;8:16833. doi: 10.1038/s41598-018-34968-6 (PMC6237991; doi:10.1038/s41598-018-34968-6)
Supplement: Supplementary file 1 — Supplementary data [file 41598_2018_34968_MOESM1_ESM.pdf]

Safe and efficient novel approach for non-invasive gene electrotransfer to skin

Lise Pasquet *, Sophie Chabot *, Elisabeth Bellard , Bostjan Markelc , Marie-Pierre Rols , Jean-Paul Reynes , Gérard Tiraby , Franck Couillaud , Justin Teissie , Muriel Golzio

Supplementary data :

S1:

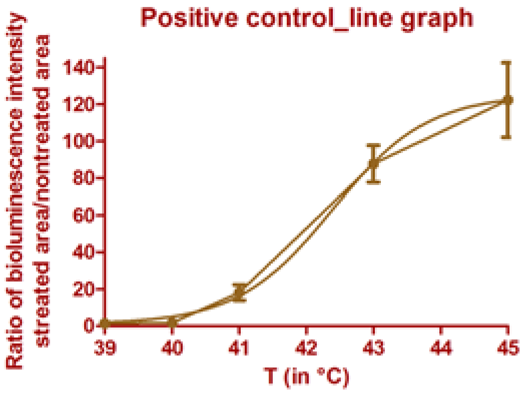

Supplementary Data 1: Positive control of the thermal effect by Quantification of Hsp70 LucF expression in transgenic mice that were treated in a water bath at designated temperature for 8 min.

S2:

A B

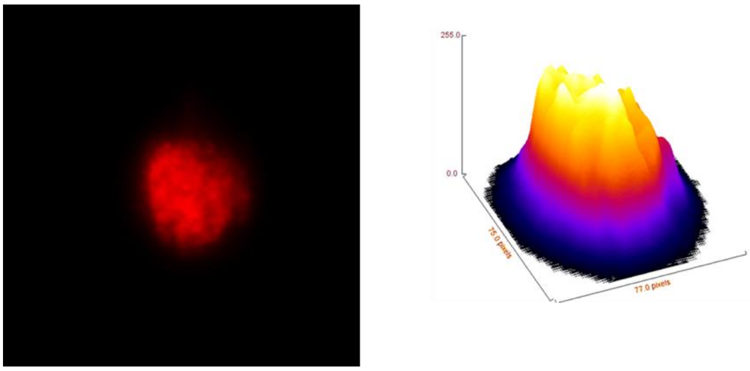

Supplementary Data 2: Direct imaging of tdTomato expression in the skin 1 day after GET. (A) Direct observation, (B) Surface plot after image analysis under Image J with a pseudo color representation. Skin was treated by 4 trains of pulses of combination of HV-MV (100 µs, 400 V +20 ms, 100 V).
